# Study of Thymidylate Synthase (TS) and Dihydropyrimidine Dehydrogenase (DPD) Expressions on 5-Fluorouracil in Oral Squamous Cell Carcinoma

**DOI:** 10.31557/APJCP.2019.20.2.503

**Published:** 2019

**Authors:** Khaleda Akhter, Mohammed Enamur Rashid

**Affiliations:** 1 *Department of Periodontology and Oral Pathology, (Division of Oral Medicine), Pioneer Dental College and Hospital, Dhaka University, Dhaka, Bangladesh,*; 2 *Department of Oral Basic and Clinical Sciences, (Division of Oral Biology), College of Dentistry, Taibah University, Al Medina Al Munawara, Kingdom of Saudi Arabia. *

**Keywords:** Cancer, chemotherapy, dihydropyrimidine dehydrogenase, prognosis, thymidylate synthase, 5-fluorouracil

## Abstract

**Background:**

The study aims to analyze Thymidylate Synthase (TS) and Dihydropyrimidine Dehydrogenase (DPD) Expressions on 5-Fluorouracil in Oral Squamous Cell Carcinoma (OSCC).

**Methods:**

50 oral squamous cell carcinoma samples were taken from non-treated cancer patients at Hiroshima University Dental Hospital. The patients were investigated for TS, that included 36 males and 14 females. Additionally, 31 patients were evaluated for DPD that included 22 males and 9 females.

**Results:**

The samples had also undergone clinical and pathological evaluation, immunohistochemical staining, evaluation of immune-staining, enzymatic expression, and statistical analyses. Mean age of the population was 62.1 years.

**Conclusion: O:**

ver-expression of TS contributes significantly to the resistance of 5-FU treatment; while inhibition of intra-tumoral DPD increases the sensitivity level. TS levels are not only predictive of 5-FU response, but also prognostic in clinical value of non-treated cancer patients.

## Introduction

5 – Fluorouracil (5-FU) is an anti-cancer drug that is prescribed against malignancies as a single therapy or combined treatment (Yagiz et al., 2015). The drug is highly preferred in the management strategies of oral squamous cell carcinomas or OSCC (Regezi et al., 2016). The efficacy of 5-FU depends on its conversion activity that modifies the abilities of an associated cell into active metabolites. 5-FU is activated in the body by processing through a three-step enzymatic cascade, where it is phosphorylated into 5-FU nucleotides (Derissen et al., 2016).

When 5-FU is administered to the patient, it converts in vivo to fluorodeoxyuridine-5-monophosphate (FdUMP) by the action of thymidine phosphate (TP), present in the cells of a tumor. FdUMP forms a stable and tight binding ternary complex with thymidylate synthase (TS) and 5, 10-methylenetetrahydrofolate. Di-hydro-pyrimidine dehydrogenase (DPD) is responsible for the breakdown of 5-FU to 5-fluoro-di-hydro-uracil (FUH2) (Etienn et al., 1995). Approximately 85% of administered 5-FU in the body is degraded through catabolic pathway, followed by this enzyme (Ikeguchi et al., 2001). Etienne et al., (1995)further determined tumoral/non-tumoral DPD activity ratio (normalized DPD) in biopsy specimens from the head and neck cancers. The complex inhibits and causes methylation of deoxyuridine-5-monophosphate (dUMP) to deoxythymidine-5- monophosphate (dTMP) by TS. This inhibitory mechanism leads to the suppression of DNA synthesis in the malignant cells (Peters et al., 1995), which ultimately suppress the expression of cancer (Almqvist et al., 2016). Fluorouridine-mono-phosphate (FUMP) is converted by the action of enzyme orotate phosphor-ribosyl transferase (OPRT) that is subsequently phosphorylated into fluorouridine-tri-phosphate (FUTP) (Peters et al., 1986). The results reported that the patients, who had responded to the study, exhibited significantly lower normalized DPD values as compared to partial and non-responding patients. Thus, DPD activity is known to be a potential factor, controlling 5-FU sensitivity of cancer cells (Vasiljević et al., 2014). Elevated intra-tumoral DPD activity has been implicated in low anti-tumor activity of 5-FU due to increased 5-FU inactivation (Jakobsson et al., 1973; Yamashita et al., 2014; Krishnan et al., 2014). FUTP is also incorporated into RNA instead of the uridine-tri-phosphate (UDTP) and interferes with the maturation and the function of RNA in the tumorous cells (Offer et al., 2014).

Evaluation of characteristics of thymidylate synthase (TS) and dihydropyrimidine dehydrogenase (DPD) on 5-Fluorouracil in oral squamous cell carcinoma would help the clinicians and researchers to develop new anti-cancer techniques that can be effective dealing with malignancies. The study has effectively investigated the response of thymidylate synthase (TS) and dihydropyrimidine dehydrogenase (DPD) to 5-fluorouracil (5FU) among OSCC patients. The study outcomes predicted that the overexpression of TS is associated with 5FU resistance. Therefore, the study has majorly contributed to the fields of surgical oncology and pathology.

## Materials and Methods

*Specimen*

The study has employed a quantitative approach to conduct a prospective study. 50 samples of oral squamous cell carcinoma were obtained in between January 2016 and September 2016 from Hiroshima University Dental Hospital. 

*Inclusion and Exclusion Criteria*

The patients, who had never taken any prior treatment and were being treated for the first time were included in the study. Patients, who had received treatment for any primary disease within 12 months before conducting the study were excluded. 

*Clinical and pathological evaluation*

Histological examination was performed on the obtained samples for clinical and pathological evaluation. The tissues were extracted using obwegeser retractor by laboratory assistants, and appropriate medication was provided after the extraction. The patients were uniformly treated, and 9 parameters were considered for the observation that included:

i. Age

ii. Gender

iii. Histologic grade

iv. Tumor size

v. Stage

vi. Lymph node metastasis

vii. Mode of invasion

viii. Recurrence of the primary tumor

ix. Survival rate

Histologic grade, lymph node metastasis, and tumor size have been classified according to the classification, provided by World Health Organization. It is essential to mark that the presently offered therapeutic modality to patients with OSCC is affirmed on the histological grading and conventional stage predicted indices (Nakamura et al., 2014) Moreover, mode of invasion has been classified by the criteria, provided by Jakobsson et al., (1973) Mode of invasion was presented with well-defined borderline, diffusion of growth, and groups of the cell with no distinct borderline (Ligabue et al., 2012). The patients were called for follow-up after a month. 

*Immuno-histochemical staining for TS and DPD*

The evaluation for immunohistochemistry of TS and DPD was performed by using anti-recombinant human TS (RTSSA) and DPD (RDPDPA) polyclonal antibodies. All immunohistochemical examinations were carried out using tissue sections from formalin-fixed, paraffin-embedded specimens. Anti-recombinant human TS (RTSSA) and DPD (RDPDPA) polyclonal antibodies were taken from the tissue specimen. 

**Table 1:** Histo-Chemical Grading with Levels of Thymidylate Synthase (TS) with Chemical Expression in Oral Squamous Cell Carcinoma

Histo-chemical Grades	High Levels of TS N (%)	Low Levels of TS N (%)
Grade I	5 (28%)	8 (62%)
Grade II	14 (40%)	21 (60%)
Grade III	2 (100%)	0 (0)

Okabe et al. (2000)

**Table 2:** Tumor Sizes with Levels of Thymidylate Synthase (TS) with Chemical Expression in Oral Squamous Cell Carcinoma

Tumor Size Grades	High Levels of TS N (%)	Low Levels of TS N (%)
T1	4 (36%)	7 (64%)
T2	2 (2%)	16 (89%)
T3	13 (87%)	2 (13%)
T4	4 (67%)	2 (33%)

Okabe et al. (2000)

**Figure 1:**
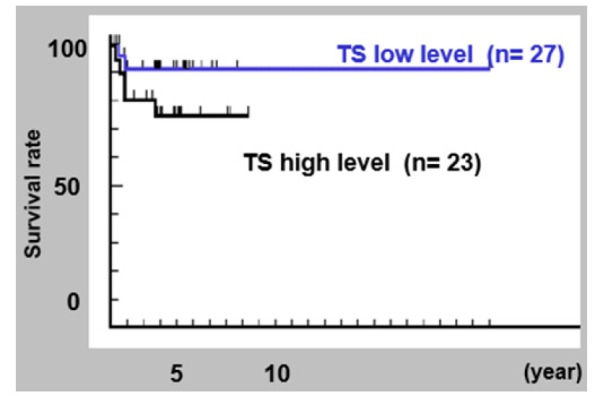
Kaplan-Meier Survival Rates among the OSCC Patients

**Figure 2:**
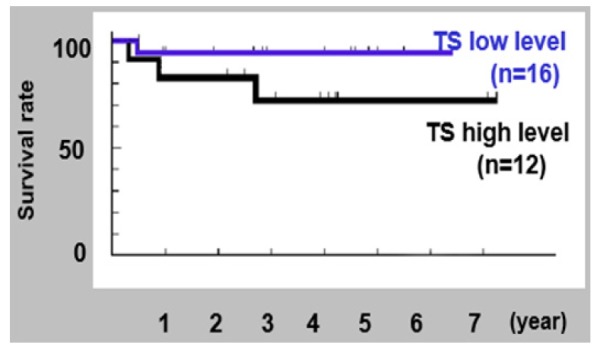
Kaplan-Meier Survival Rates of Tongue among the Cancer Patients

**Table 3:** Chemotherapeutic Responses with Chemical Expression in Oral Squamous Cell

Chemical Expression	Response (N)	No Response (N)
Thymidylate Synthase (TS)	24 (48%)	26 (52%)
Dihydro-Pyrimidine Dehydrogenase (DPD)	27 (87%)	4 (13%)

**Table 4:** Five Years Survival Rate with Chemical Expression in Oral Squamous Cell Carcinoma

Chemical Expression	High Levels (%)	Low Levels (%)	P - value
Thymidylate Synthase (TS)	75.70%	92.60%	0.04
Dihydro-Pyrimidine Dehydrogenase (DPD)	72.90%	93.80%	0.05

*Immunohistochemical staining procedure for TS*

A section of 4 μm thickness was obtained. The samples were deparaffinized in xylene and rehydrated with gradual graded alcohols. Endogenous peroxidase activity was blocked by soaking sections in 0.3% hydrogen peroxide for 20 minutes. For antigen retrieval, slides were heated twice at 98^o^C for 10 minutes in an oven and then cooled for 20-30 minutes at room temperature. All sections were placed in 10 mM citrate buffer solution (pH 6.0), after washing them with tap water, subsequently followed by distilled water. After washing the samples twice at 4^o^C in Dulbecco’s phosphate buffered saline (PBS), non-specific reactions were blocked by pre-incubation for 30 minutes at 4^o^C, with normal goat serum (Vector Laboratories, Inc, CA, USA).

These sections were further incubated at 4^o^C overnight by aforementioned primary antibody, in which the optimal concentration was kept 1:1,000. Slides were incubated with biotinylated anti-rabbit immunoglobulin in PBS, for 30 minutes after washing the samples thrice in PBS at 4^o^C, at room temperature. The samples were again washed thrice in PBS, at 4^o^C, followed by incubation with Elite ABC solution (Vector Labcs. Inc. CA, USA). The washing procedure was repeated. The immunochemical reaction was detected by using DAB Substrate-Chromogen system (Dako Corp., CA USA), and was stopped after 3-5 minutes. All sections were briefly counterstained with Mayer’s hematoxylin and then mounted after washing with tap water, followed by distilled water.

*Immuno-histochemical staining procedure for DPD*

The procedure is similar to TS. Sections of 4 μm thickness were sliced, deparaffinized in xylene, and rehydrated subsequently with graded alcohols. The sections were then incubated overnight with aforementioned primary antibody at 4^o^C; the optimum concentration has been kept 1:500. After washing with PBS at 4^o^C for 60 minutes, the sections were incubated with biotinylated anti-rabbit immunoglobulin in PBS for 30 minutes. The sections were then washed with PBS at 4^o^C for 30 minutes and incubated again in Elite ABC solution (Vector Laboratories, Inc, CA, USA). Subsequently, the washing manipulation was carried out for 60 minutes at 4^o^C. The immunochemical reaction has been evaluated by using DAB Substrate-Chromagen system (Dako Corp., CA, USA). The reaction has been stopped after 3-5 minutes. The sections were counterstained with Mayer’s hematoxylin and then mounted, after washing with tap water and followed by distilled water.

*Evaluation of immune-staining*

*Evaluation of TS expression*

TS expression was scored semi-quantitatively by using the percentage of stained cells in each tumor. At least, five fields were observed and the numbers of stained cells were counted. In each field, at least 200 cells were counted. TS expression was divided into two groups based on the grade of staining:

• Low staining group was without the staining of more than 50% cancerous cells.

• High staining group had more than 50% cells stained.

Human tumor xenograft was implanted into a nude mouse DLD-1/FdUrd. The animal has been modelled as a positive control for TS. The negative control has been developed by using non-immunized rabbit immunoglobulin G (IgG), instead of the primary antibody.

*Evaluation of DPD expression*

DPD staining has also been expressed semi-quantitatively as stained cell percentage out of a total number of tumor cells, and assigned to four gradings, mentioned as follow (Okabe et al., 2000):

Grade 1: <25% positive cells in staining,

Grade 2: >25% and <50% positive cells,

Grade 3: >50% but <75% positive cells in staining,

Grade 4: >75% positive cells.

Tumor tissue from xenograft of human pancreatic cancer cell line, MIAPaCa-2 was implanted in the nude mouse as the positive control. The negative control has been developed by using non-immunized rabbit IgG instead of the primary antibody.

*Statistical analyses*

The data, obtained from the observation, was analyzed with Mann-Whitney’s U test and Spearman’s rank correlation test. As for TS, survival curves were estimated by Kaplan-Meire method, and their differences were evaluated by Mantel-Cox test. P-values of <0.05 were considered as significant.

## Results

*Patients’ characteristics*

Fifty non-treated cancer patients have been tested for thymidylate synthase (TS), that included 36 males and 14 females. As for Di-hydro-pyrimidine dehydrogenase (DPD), 31 patients were examined amongst whom 22 were males and 9 were females. The mean age of the study population had been 62.1 years.

*Immunohistochemical expressions of TS and DPD*

In cancer tissues, TS staining patterns have shown variables within and amongst individual tumors. Undifferentiated tumor cells at the epithelial-connective tissues interface showed granular cytoplasmic staining, while terminally differentiated cells at the center of the nest did not express any staining. Histochemical grading has been presented in grades, in Table 1.

*Clinicopathologic parameters and expressions of TS and DPD*

The study group expressing high TS had been observed to have more advanced tumors than the group with low TS (P = 0.0023). It has been revealed that significant difference between the two groups depends on the tumor size. No significant correlation was observed between TS expression and the other clinicopathologic parameters. Tumor sizes have been recorded in grades and presented in Table 2. The results have shown that majority of the patients (87%) were suffering from third grade tumor with high levels of TS. While, 89% of them were suffering from second grade tumor with low levels of TS.

*Chemotherapeutic response and expression of TS and DPD*

The study observed no change in TS immunoexpression before and after chemotherapy among 5 patients; whereas, 3 patients had evidently expressed the change. Chemotherapy was chosen as the first mode of therapy for oral squamous cell carcinoma because of reduced extent of surgical resection, decreased distant metastasis, and improved loco-regional control. All of these factors improve the treatment outcomes by decreasing the rates of morbidity and mortality. There were 15 respondents of the therapy. Out of them, 10 patients showed partial chemo-response, amongst whom 3 patients had low levels of TS while 7 had high levels of TS. Furthermore, there were 5 non-responders, amongst whom 3 patients had low levels of TS while 2 had high levels of TS. Moreover, 13 other cases were witnessed amongst whom 9 patients expressed partial response to chemotherapy. The grading for DPD categorized them into the frequency of 6, 2 and 1 for Grade 1, Grade 2 and Grade 3, respectively; and the remaining were of Grade 4 category. However, 4 patients did not respond to the therapy in any notable manner. The incidence of response with regard to the chemical expressions has been presented in Table 3.

*Univariate analysis of survival rate*

The survival rate of five years had been observed in 84.4% of the study population. The rates in the low and high TS group were noted to be 92.6% and 75.7%, respectively. No statistical difference was seen in the survival rate between these two groups. The survival rate of low TS group was significantly better than that of high TS group (P = 0.040), in the case of tongue carcinomas. Five-year survival rates in low and high DPD groups were observed to be 93.8% and 72.9%, respectively. The record for survival rate with regard to the chemical expressions has been presented in Table 4. Figures 1 and 2 display the survival rates amongst the patients of OSCC and tongue cancers. 

## Discussion

This study employed the immunohistochemical technique to examine TS and DPD expressions among OSCC patients. Cumulative evaluation of TS and DPD on oral squamous carcinoma cell lines was observed to offer prognostic value, concerning the clinicopathological significance of 5 fluorouracil. A similar tendency was observed in relation between TS expression and tumor stage. Four cases have been identified that were exhibiting low TS levels. They were classified into stage IV due to nodal involvement. A similar trend was seen between TS expression and recurrence of primary tumor. However, the correlation did not express any significance (P = 0.1029). It is reasonable that these parameters showed a significant correlation or similar relationship with TS expression, which is one of the prominent indicators of cell proliferation. TS was inhibited by 5-FU treatment that was caused by imbalances in nucleotide pool of the tumor cells, which inhibited the synthesis of DNA. There have been several reports that have presented high expression of TS in correlation with poor prognostic determinations and resistance to 5-FU based chemotherapies. Predominantly, the cells that express resistance towards 5-FU showed high TS expressions (Siddiqui et al., 2017). 

There is a statistically significant correlation between TS expression and tumor size; however, no correlation was seen with other parameters. In prognosis, there has been no significant difference noted in the overall survival rate between TS high and low level. However, the survival curve for low TS group is better for high TS group in tongue carcinoma patients. A large scale of study is required to conclude because the number of recurrent cases was smaller in the present study. As per previous researchers and clinical studies, large-scale evaluations can significantly contribute in determining the prognosis of the disease. It can further assist in choosing treatment options with regard to chemical profiling of cancerous cells; thus, adding benefits to the quality of life of the patient. Based on tumor sizes, 13 (87%) of 15 T3 cases and 4 (66%) of 6 T4 cases were observed in high TS levels. T4 cases might have stopped growing or reached the phase of necrosis due to poor nutrition following the overgrowth of the tumor (Morimoto et al., 2006). On the other hand, low TS level was observed in 16 of 18 T2 cases (89%) and 7 of 11 T1 cases (64%), respectively. The above results may suggest that most T3 tumors have been in proliferative stage; whereas, most T2 tumors have been in less active stage (Yamamoto et al., 1983).

Immunopositivity was generally observed in undifferentiated cells of the basal layer at the epithelial-mesenchymal interface. In cases of high TS level, immunopositivity was heterogeneously observed in differentiated cells. 21 cases (60%) showed low TS level in histological Grade II, while 8 (62%) of 13 cases showed high TS level in Grade I. Grade III was observed in only two cases. There was no statistical correlation found between TS level and histologic grade. Most of the invasion front cells, mainly consisting of undifferentiated cells, were found to be immunopositive. There has been no relationship found between the levels of TS and mode of invasion that might have been associated with metastasis, local recurrence or prognosis (Zhao et al., 2013). With regard to prognosis, there has been no statistical difference in the overall survival rate between TS low level and TS high level. However, the survival rate of low TS group has been significantly better than that of high TS high group in tongue cancer patients (P = 0.040). One of the reasons responsible for this association is tumor size relating to TS level of the main prognostic factors in tongue cancers. As for DPD, there has been no correlation found between the DPD expression and clinicopathologic parameters, even though a small number of patients were examined. Furthermore, TS and DPD expressions did not correlate with chemo-response in patients (Tanaka et al., 2012).

It has been investigated and claimed through several types of research that low expression of TS serves a predicting factor for high reactivity towards chemotherapy that can eventually improve survival rates (Bai et al., 2015). Studies have further highlighted that levels of TS may not accurately present the prognostic values for tumors and chemotherapeutic efficacy (Sadahiro et al., 2012). However, it has also been presented that high levels of TS may deliver the probability of poor prognostic features (Yamada et al., 2008). Other studies have indicated that high levels of TS can be associated to therapeutic features, while low levels can be useful to predict the prognosis of the disease and plan the treatment strategies. A study evaluated the correlation between the curative influences of 5 fluorouracil based chemotherapy and presented a negative association between the two entities. It has been seen that the therapeutic effects decrease with the increasing expression of DPD, eventually reducing the survival probability of the patients (Yamada et al., 2008). A study conducted by Kikuchi et al. (2015) stated that 5-FU resistance of TE-5R cells developed as a result of rapid degradation of 5-FU by DPD overexpression. The amplification and consequent DPD overexpression is likely to generate novel biological evidence for exploring strategies against ESCC with 5-FU resistance. Another study conducted by Yoshida et al., (2015) revealed that mortality rate associated with chemotherapy is high among DPD-deficient patients. Moreover, there is no reporting about the continuation of chemotherapy among the patients with DPD activity below 10%. study has several limitations, including the heterogeneous sample size, which was too small due to lack of time and extensive procedure. IHC-based approaches were used as descriptive and subjective in nature, and no functional studies were performed.

In conclusion, the present study has determined that chemical modalities can be used to create predictive forms regarding therapeutic alternatives. In the current study, the immunohistochemical technique has been performed in OSCC samples. This technique is easy to perform and applicable on formalin-fixed tissue. Furthermore, it has highlighted its role in determining the efficacy of TS and DPD, and enhancing the survival rate of patients, undergoing the preferred treatment. The over-expression of TS plays a major role in the resistance to 5-FU treatment, while inhibition of intra-tumoral DPD increases sensitivity. It has been elucidated that TS levels are not only predictive of 5-FU response, but also prognostic in clinical value of non-treated cancer patients. The future studies may execute the process on larger sample size and must confirm the existing observations.

## Conflict of Interests

The author declares no competing interests. 
